# Impact of Mothers’ Schistosomiasis Status During Gestation on Children’s IgG Antibody Responses to Routine Vaccines 2 Years Later and Anti-Schistosome and Anti-Malarial Responses by Neonates in Western Kenya

**DOI:** 10.3389/fimmu.2018.01402

**Published:** 2018-06-18

**Authors:** Bartholomew N. Ondigo, Erick M. O. Muok, John K. Oguso, Sammy M. Njenga, Henry M. Kanyi, Eric M. Ndombi, Jeffrey W. Priest, Nupur Kittur, William Evan Secor, Diana M. S. Karanja, Daniel G. Colley

**Affiliations:** ^1^Centre for Global Health Research, Kenya Medical Research Institute (KEMRI), Kisumu, Kenya; ^2^Department of Biochemistry and Molecular Biology, Egerton University, Nakuru, Kenya; ^3^Eastern and Southern Africa Centre of International Parasite Control, Kenya Medical Research Institute (KEMRI), Nairobi, Kenya; ^4^Department of Pathology, Kenyatta University, Nairobi, Kenya; ^5^Division of Foodborne, Waterborne, and Environmental Diseases, Centers for Disease Control and Prevention, Atlanta, GA, United States; ^6^Center for Tropical and Emerging Global Diseases, University of Georgia, Athens, GA, United States; ^7^Division of Parasitic Diseases and Malaria, Centers for Disease Control and Prevention, Atlanta, GA, United States; ^8^Department of Microbiology, University of Georgia, Athens, GA, United States

**Keywords:** schistosomiasis, malaria, antibody responses, tetanus, diphtheria, measles, vaccines

## Abstract

The potential consequences of parasitic infections on a person’s immune responsiveness to unrelated antigens are often conjectured upon in relationship to allergic responses and autoimmune diseases. These considerations sometimes extend to whether parasitic infection of pregnant women can influence the outcomes of responses by their offspring to the immunizations administered during national Expanded Programs of Immunization. To provide additional data to these discussions, we have enrolled 99 close-to-term pregnant women in western Kenya and determined their *Schistosoma mansoni* and *Plasmodium falciparum* infection status. At 2 years of age, when the initial immunization schedule was complete, we determined their children’s IgG antibody levels to tetanus toxoid, diphtheria toxoid, and measles nucleoprotein (N-protein) antigens using a multiplex assay. We also monitored antibody responses during the children’s first 2 years of life to *P. falciparum* MSP1_19_ (PfMSP1_19_), *S. mansoni* Soluble Egg Antigen (SEA), *Ascaris suum* hemoglobin (AsHb), and *Strongyloides stercoralis* (SsNIE). Mothers’ infections with either *P. falciparum* or *S. mansoni* had no impact on the level of antibody responses of their offspring or the proportion of offspring that developed protective levels of antibodies to either tetanus or diphtheria antigens at 2 years of age. However, children born of *S. mansoni*-positive mothers and immunized for measles at 9 months of age had significantly lower levels of anti-measles N-protein antibodies when they were 2 years old (*p* = 0.007) and a lower proportion of these children (62.5 vs. 90.2%, OR = 0.18, 95% CI = 0.04–0.68, *p* = 0.011) were considered positive for measles N-protein antibodies. Decreased levels of measles antibodies may render these children more susceptible to measles infection than children whose mothers did not have schistosomiasis. None of the children demonstrated responses to AsHb or SsNIE during the study period. Anti-SEA and anti-PfMSP1_19_ responses suggested that 6 and 70% of the children acquired schistosomes and falciparum malaria, respectively, during the first 2 years of life.

## Introduction

There is a concern that children in developing countries do not respond appropriately to standard vaccines given through the expanded program of immunization (EPI), and this is most often discussed in regard to the mother’s parasitic disease status during gestation. Emerging evidence suggests that some chronic antenatal helminth infections may alter infant immune responses to immunization with Bacille Calmette–Guérin (BCG), *Haemophilus influenzae*, measles, and polio vaccines (1–4). It is even possible that this influence may last for decades (5, 6). Since vaccine-preventable diseases account for an estimated two to three million deaths in African children each year (7), the extent to which parasitic diseases during gestation influence EPI vaccine effectiveness in neonates could have critical public health consequences.

Although treatment of pregnant women for schistosomiasis is recommended by the World Health Organization (WHO) (8–10), in many countries pregnant women continue to be excluded from mass drug administration (MDA) programs because of unfounded concerns about potential effects of praziquantel on the developing fetus (11). If the helminth infection status of a pregnant woman decreases the effectiveness of current and/or future vaccine responses of her newborn, anti-helminthic treatment during pregnancy would take on an additional level of public health importance.

This study sought to understand whether being born of a schistosome-infected mother altered the antibody response levels of her child at 2 years of age to the standard vaccines against tetanus, diphtheria, and measles given through the Kenya Expanded Program of Immunization (KEPI). We also evaluated the correlation of the IgG antibody levels in mothers and their neonates at birth to antigens from the following parasites: *Schistosoma mansoni, Plasmodium falciparum, Ascaris lumbricoides*, and *Strongyloides stercoralis*. In addition, we determined the levels of IgG antibodies to these parasite antigens in these children during their first 2 years of life.

## Materials and Methods

### Study Design and Study Participants

This longitudinal serologic study was conducted in western Kenya over a 4-year period, between 2013 and 2017. We enrolled late-term pregnant women and followed their children for 2 years for antibody responses to parasite antigens and standard immunizations. Pregnant women were diagnosed for malaria, soil-transmitted helminths (STH; *A. lumbricoides*; *Trichuris trichiura*, and hookworm), and schistosomiasis. Blood samples were collected from mothers, and from babies at or near birth, and subsequently until 2 years of age (see below).

Pregnant women were recruited from the antenatal clinics at several county hospitals which were selected based on the average number of pregnant women visiting the facility, nearness of the facility to the Kenya Medical Research Institute (KEMRI) in Kisian, Kenya, and to represent multiple categorical levels of the hospitals. The six study hospitals used were: Jaramogi Oginga Odinga teaching and referral hospital (the largest facility in Kisumu offering comprehensive services in the region—level 5), Kisumu sub-county hospital (level 4), Port Florence and Ober Kamoth health centers (level 3), Usoma and Rota dispensaries (level 2). All the facilities used are government funded except Port Florence, which is private. Pregnant women who were in their third trimester (34–37 weeks gestation) were screened for eligibility and asked to participate in the study. Those who consented filled out a questionnaire and donated a single blood sample and three stool specimens collected on consecutive days. The name, detailed address, and phone number of each consented participant was recorded at the time of enrollment to facilitate home based follow-up of their child until 24 months postpartum. Each woman was assigned a unique study number that was then consistent with that of her child throughout the study. Pregnant women who were smear-positive for malaria were treated according to Kenya Ministry of Health guidelines. Mothers infected with schistosomes or intestinal helminths were treated soon after delivery.

The objective of the study was explained in detail to all enrolled pregnant mothers in the local language (Luo) in the presence of community midwives who were not part of the study. Witnessed written informed consent was obtained from each subject for themselves and for their unborn child to participate over the entire 2-year follow-up period. A copy of the signed consent form was given to each enrollee and another copy was kept in a locked cabinet with restricted access in the offices of the KEMRI, Centre for Global Research at Kisian, in Kisumu County. Only infants born at term, defined as a gestational age of 37–42 weeks, were included in this study. When twins were born, both were enrolled in the study. Before birth, each mother was confirmed to have been provided with a vaccination clinic booklet that was to be used to record and track vaccination history of the infant and the health status of both the mother and child. In accordance with KEPI, infants were vaccinated with BCG vaccine at birth; 3 doses of [diphtheria, pertussis, tetanus (DPT)] vaccine, at 6, 10, and 14 weeks; oral polio vaccine (OPV) at or within 2 weeks of birth and 6, 10, 14 weeks; and measles vaccine at 9 months. BCG, DPT, OPV, and measles vaccines, all of which were recommended and pre-qualified by WHO, were manufactured by Serum Institute of India PVT, Hadapsar, India. Vaccine manufacturers provided the study product but had no role in the conduct of the study, analysis of the data, or preparation of this report. These vaccines were provided free-of-charge by the Ministry of Health officials through the Division of Vaccines and Immunization under KEPI. All immunizations were recorded on an infant birth vaccination clinic booklet brought by the mother and all dates of vaccinations were verified for all children by our field study team.

### Ethical Statement

The research protocol was approved by the Scientific Steering Committee of the Kenya Medical Research Institute (SSC-KEMRI), KEMRI/Scientific Ethical Review Unit (Protocol No. 2303) and the Institutional Review Board at the University of Georgia (Protocol No. 00004091). The Centers for Disease Control and Prevention (CDC) also reviewed the protocol; CDC personnel were considered not to be engaged because they had no contact with study participants or access to personal identifiers. All subjects provided written informed consent in accordance with the Declaration of Helsinki.

### Blood Collection and Processing

Maternal venous blood was collected at the time of the neonate’s first bleed, at or a few days after birth. Subsequent blood collections from children were at 6 and 20 weeks and 9, 12, 18, and 24 months of age. At least 400 µL of heel prick or finger blood was collected by capillary blood collection using lithium heparin anticoagulant (Kabe Labortechnik GMBH, Germany). Blood was centrifuged to isolate the plasma fraction from the cell pellet and plasma specimens were stored at −20°C. Antibody assays were performed at the same time after all plasmas were collected.

### Diagnosis of Schistosomiasis, STHs, and Malaria

Detection and enumeration of *S. mansoni* eggs were determined by Kato Katz fecal examination (12) based on two slides from each of the three stool samples collected from women upon enrollment. Intensity of infection was obtained for *S. mansoni* as eggs per gram of feces (epg) and the presence or absence of eggs of the three STH was recorded. Blood from all pregnant women was examined for malaria parasites. Thick and thin blood smears were prepared, stained with 10% Giemsa for 15 min, and examined by light microscopy for *Plasmodium*-infected erythrocytes. At least 200 microscope fields were scanned before a smear was considered as negative. Children were not assayed for malaria parasites in their blood, nor were they evaluated by stool examination for schistosome infection.

### IgG Antibody Levels Measured by Luminex Multiplex Bead Assay

Fluorescent bead–based multiplex bead immunoassays were performed using Luminex technology (Luminex Corp., Austin, TX, USA) to analyze plasma antibody levels to multiple antigens from mothers and infants. Expression and purification of *S. stercoralis* NIE (SsNIE) (13) and *P. falciparum* 19-kDa subunit of Merozoite Surface Protein 1 (MSP1_19_) (14) antigens as fusion proteins with *Schistosoma japonicum* glutathione-S-transferase (GST) have been described (15, 16). Recombinant GST with no fusion partner was also expressed and purified as previously described (17) for use as a negative control in the multiplex assays. The conditions for coupling these antigens to the beads have been described (16) as has coupling of *S. mansoni* soluble egg antigen (SEA) (18). Native hemoglobin *Ascaris suum* hemoglobin (AsHb) purified from *A. suum* worms was a kind gift of Peter Geldhof (Ghent University, Belgium) (19, 20). SEA and AsHb were coupled to SeroMap microsphere beads (Luminex Corp., Austin, TX, USA) in phosphate-buffered saline (PBS) at pH 7.2 using 120 µg protein for 12.5 × 10^6^ beads as previously described (18).

The following antigens were purchased from commercial sources: tetanus toxoid (Massachusetts Biological Laboratories, Boston, MA, USA), diphtheria toxoid from *Corynebacterium diphtheriae* (List Biological Laboratories, Campbell, CA, USA), and recombinant measles nucleoprotein (MV-N, Meridian Life Sciences, Memphis, TN, USA) (21). Tetanus toxoid was coupled to SeroMap beads as previously described (16). Diphtheria toxoid was coupled in buffer containing 50 mM 2-*N*-morpholinoethanesulfonic acid (MES) at pH 5.0 with 0.85% NaCl using 60 µg of protein per 12.5 × 10^6^ beads in 1 mL final volume. Measles nucleoprotein (N-protein) was partially purified by MonoQ column chromatography (GE Healthcare, Piscataway, NJ, USA) and coupled to beads in buffer containing 50 mM MES/NaCl buffer at pH 5.0 using 6 µg of protein per 12.5 × 10^6^ beads in 1 mL final volume.

Test plasma samples were diluted 1:400 in PBS buffer (pH 7.2) containing 0.3% Tween 20, 0.02% sodium azide, 0.5% casein, 0.5% polyvinyl alcohol, 0.8% polyvinylpyrrolidone, and 3 µg/mL *Escherichia coli* extract (15, 18, 22). Multiplex bead assays for total IgG antibodies were performed as previously described (15, 23). Each assay plate included a buffer only blank, as well as positive and negative controls, and all samples were tested in duplicate. Data were collected using a BioPlex 200 instrument with BioPlex Manager version 6.1.1 software (BioRad, Hercules, CA, USA). Responses are reported as the average of the median fluorescent intensity minus background for the duplicate wells (MFI-BKG). Samples having a coefficient of variation of >15% between the duplicate wells for >3 positive antibody responses were repeated. All samples collected from one child were assayed on the same plate to minimize potential impacts caused by variability in assay performance.

Cutoffs for the responses to the vaccine antigens were determined using reference standards TE-3 (tetanus) and 10/262 (diphtheria) purchased from National Institute for Biological Standards and Control in the Hertfordshire, UK. Twofold serial dilutions were performed, and a log–log plot used to fit a line to the data below the response plateau. Correlations were all consistently high. Median fluorescence intensity (MFI) cutoffs were as follows: diphtheria cutoff for complete protection = 0.1 IU/mL = 4,103 MFI-BKG (24, 25); tetanus cutoff for protection = 10 mIU/mL = 43 MFI-BKG (16, 26). The choice of measles N-protein assay cutoff was based on a receiver-operating characteristic curve analysis of 140 sera comparing multiplex bead assay to the “gold standard” plaque reduction neutralization assay, a live virus infection assay that measures all classes of virus-neutralizing immunoglobulin. The 149 MFI-BKG cutoff value calculated at the CDC in Atlanta, GA, USA, was translated to the data generated at KEMRI using a twofold serial dilution standard curve that was run at both locations. The resulting measles N-protein assay cutoff for the KEMRI BioPlex 200 instrument was 67 MFI-BKG units.

The optimal cutoff points for positive antibody levels against PfMSP1 were determined by assigning all mothers’ responses in this holoendemic area as positive and all children’s responses at 20 weeks of age as negative and calculating an ROC curve and J-Index (GraphPad Prism version 6 for Windows, San Diego, CA, USA). The cutoff for anti-SEA antibodies was determined by using the mixture model reported in Cutoff Finder using R (27).

### Development of the Final Dataset for Analysis

The final dataset used for analysis was comprised of mother/neonate pairs for which the mother and her neonate were concordant for either presence or absence of anti-SEA IgG and for mother/neonate pairs where the mother’s plasma contained anti-SEA IgG and her stool specimens were positive for *S. mansoni* eggs. Egg-negative mothers were Kato Katz-negative but could be anti-SEA IgG antibody negative or positive with a consistent antibody response in the neonate. Mothers in this category were assumed either to be: (a) uninfected; (b) previously infected; or (c) infected with low eggs per gram feces below the Kato Katz limit of detection. Mother/neonate pairs for which the mother and her neonate were discordant for the presence of any anti-SEA IgG (i.e., one member of the pair had specific antibody and the other was completely negative) were deemed to be mistakenly matched during data collection or entry and were, therefore, excluded from final analyses.

### Statistical Analysis

Data were cleaned and analyzed using IBM SPSS Statistics for Windows, Version 24.0 (IBM Corp., Armonk, NY, USA). GraphPad Prism version 6 for Windows (La Jolla California) was used for statistical analyses as well as for graph preparation. Characteristics of the participating mothers were summarized using descriptive statistics. Anemia was categorized using WHO-recommended cutoffs (28) adjusted by age and altitude with the following hemoglobin values: normal (>11.1 g/dL), mild (10.2–11.1 g/dL), moderate (7.2–10.1 g/dL), and severe (<7.2 g/dL). Correlations between a mother’s and her infant’s antibody levels at birth were examined using Spearman’s correlation test and simple linear regression. Antibody levels were log-transformed due to skewed distribution. The Mann–Whitney *U* test was used to compare vaccine antibody responses in *S. mansoni* egg positive and negative mothers. To determine whether an infant reached a protective antibody level, the following cutoffs were used: diphtheria: 4,103 MFI (mean fluorescence intensity) minus background, tetanus: 43 MFI minus background, and Measles: 67 MFI minus background. Chi square analysis was used to determine whether the proportion of vaccine-protected infants differed between *S. mansoni* egg positive and negative mothers. Tests were considered statistically significant at *p* < 0.05.

## Results

### Demographic Characteristics of the Study Participants at Enrollment

A total of 99 mother/child pairs comprised the final dataset for analyses. The mothers’ ages and other general characteristics and the status of their parasitologic infections are shown in Table 1.

**Table 1:** Characteristics of pregnant women (*n* = 99) from western Kenya enrolled in the cohort study.

Characteristic		Sm egg positive (*n* = 42)	Sm egg negative (*n* = 57)	*p*-Value
Age in years, median (range)		23 (14–36)	22 (16–39)	0.573

Gravidity, *n* (%)	1	7 (17.5)	12 (22.2)	0.852
	2	10 (25.0)	13 (24.1)	
	3+	23 (57.5)	29 (53.7)	

Anemia, *n* (%)	No anemia	16 (40.0)	12 (22.2)	0.004*
	Mild anemia	13 (32.5)	7 (13.0)	
	Moderate anemia	10 (25.0)	33 (61.1)	
	Severe anemia	1 (2.5)	2 (3.7)	

BMI, median (range)		22.8 (17.9–30.1)	22.6 (17.7–28.7)	0.531

Sm infection intensity, *n* (%)	Negative (0 epg)	–	57 (100)	N/A
	Light (1–100 epg)	27 (64.3)	–	
	Moderate (101–400 epg)	9 (21.4)	–	
	Heavy (>400 epg)	6 (14.3)	–	

Malaria smear Positive, *n* (%)		2 (5.0)	5 (9.3)	0.437

STH prevalence, *n* (%)	*Ascaris lumbricoides*	0 (0)	4 (7.4)	0.079
	Hookworm	1 (2.5)	5 (9.3)	0.185
	*Trichuris trichiura*	2 (5.0)	2 (3.7)	0.758

*Sm, Schistosoma mansoni, BMI, body mass index, STH, soil-transmitted helminthes*.

*Indicates statistical significance.

### Impact of Mothers’ Malaria or Schistosomiasis Status on Their Children’s Antibody Responses to Tetanus, Diphtheria, and Measles Vaccines at 2 Years of Age

Only seven of the pregnant women were malaria smear-positive prior to delivery (Table 1). Analyses indicated that a mother’s malaria infection had no impact on the level of antibody response levels of her child at 2 years to tetanus, diphtheria, or measles antigens (data not shown). However, because of this low number of malaria-positive mothers, likely due to relatively standard malaria treatment during their antenatal visits, we do not present analyses of antibody infants antibody levels based on maternal malaria status.

**Figure 1:**
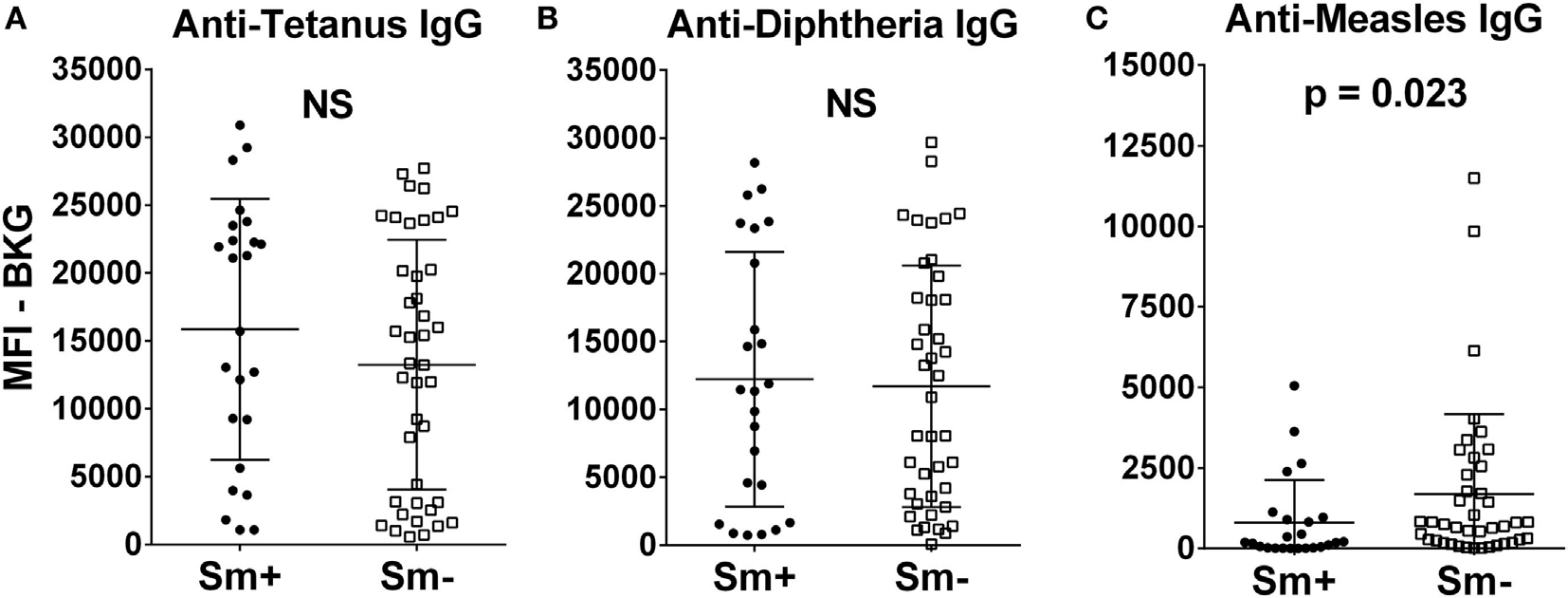
Anti-tetanus IgG **(A)**, anti-diphtheria IgG **(B)**, and anti-measles IgG **(C)** levels at 2 years of age of infants born to mothers who were either egg positive (Sm+, represented by solid circles) or egg negative (Sm−, represented by solid squares) for *Schistosoma mansoni*. All IgG responses are shown as median fluorescence intensity (MFI) minus background (BKG). Data are presented as dot plots with means at centerline, and whiskers representing the SD. Comparison was done by Mann–Whitney *U* test. NS, not significant.

**Figure 2:**
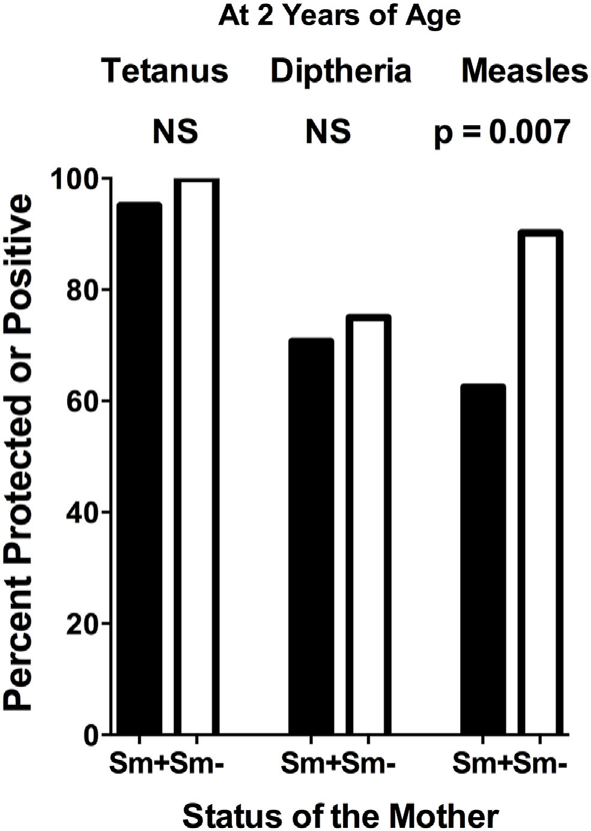
Proportion of infants at 2 years of age, born to mothers who were either egg positive (Sm+) or egg negative (Sm−) for *Schistosoma mansoni*, who had protective levels of anti-tetanus toxoid or anti-diphtheria toxoid antibodies or who were positive for anti-measles nucleoprotein antibodies in their plasma. Cutoffs to determine these levels were as follows: tetanus cutoff for protection = 10 mIU/mL = 43 median fluorescence intensity (MFI) minus background (MFI-BKG); diphtheria cutoff for complete protection = 0.1 IU/mL = 4,103 (MFI-BKG); and measles cutoff = 67 MFI-BKG units. Comparisons were done by chi square analysis. NS, not significant.

At 2 years of age, whether a child’s mother was or was not positive for *S. mansoni* eggs during the child’s gestation had no significant effect on the level of either anti-tetanus IgG or anti-diphtheria IgG (Figures 1A,B). In addition, the schistosomiasis status of mothers during their pregnancy did not impact the proportion of their children who had protective levels of anti-tetanus toxoid or anti-diphtheria toxoid antibodies in their plasma (Figure 2). However, children born of mothers with schistosomiasis exhibited significantly lower mean levels of anti-measles N-protein antibodies at 2 years of age, compared to those born of mothers with no detectable *S. mansoni* eggs in their feces during their pregnancy (Figure 1C) (*p* = 0.023). In addition, the lower anti-measles N-protein antibody levels seen at 2 years of age in children born of *S. mansoni*-infected mothers translated, based on our estimated cutoff value, to a significantly lower proportion (62.5 vs. 90.2%, OR = 0.18, 95% CI = 0.04–0.68, *p* = 0.007) of these children with anticipated protection against measles (Figure 2). Analysis of mothers’ intensities of their *S. mansoni* infections in terms of eggs per gram of feces and the responses of their children to the vaccine immunizations did not show a relationship between these parameters for any of the vaccine responses.

### Transplacental Transfer of IgG to Parasite Antigens From Mothers to Their Neonates

**Figure 3:**
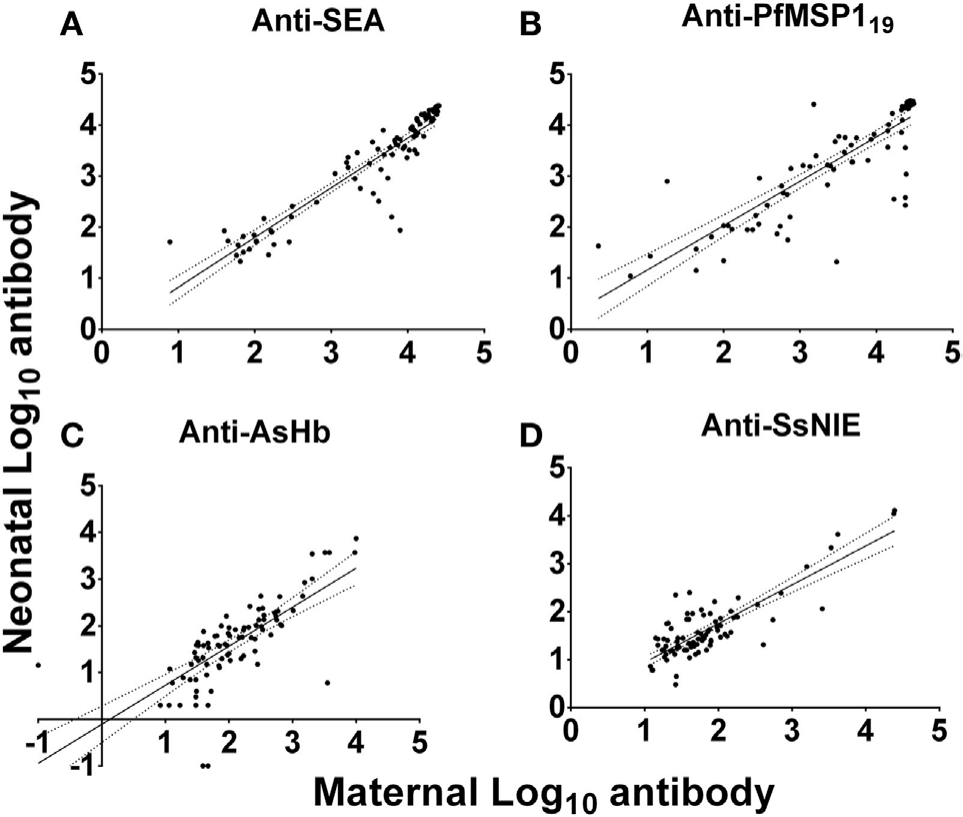
Correlation of antibody levels of mothers and their neonates to parasite antigens soluble egg antigen (SEA) **(A)**, PfMSP1_19_ **(B)**, *Ascaris suum* hemoglobin (AsHb) **(C)**, and SsNIE **(D)**. The solid line represents the regression trend line, flanked by dotted lines representing the 95% confidence interval (anti-SEA, *r* = 0.94; anti-PfMSP1_19_, *r* = 0.89; anti-AsHb, *r* = 0.80; anti-SsNIE, *r* = 0.65). All correlations were significant (*p* < 0.0001).

Anti-SEA levels in the plasmas of all mothers and their neonates correlated well (Figure 3A). However, it should be noted that these pairs were selected based on concordance of the presence of anti-SEA IgG. They were not, however, selected based on anti-SEA IgG levels. As with anti-SEA IgG, throughout the spectrum of antibody levels, mothers and their neonates shared similar anti-PfMSP1_19_ levels (Figure 3B). Although the antibody levels of mothers and their neonates were generally low to both the AsHb and SsNIE antigens, they were again correlated between pairs across the MFI spectrum (Figures 3C,D). Maternal IgG antibodies against the antigens of parasites common in western Kenya were transported effectively from mother to fetus across the placenta (anti-SEA, *r* = 0.94; anti-PfMSP1_19_, *r* = 0.89; anti-AsHb, *r* = 0.80; anti-SsNIE, *r* = 0.65) and all correlations were significant (*p* < 0.0001).

### IgG Antibody Responses to Parasite Antigens During the First 2 Years of Life

**Figure 4:**
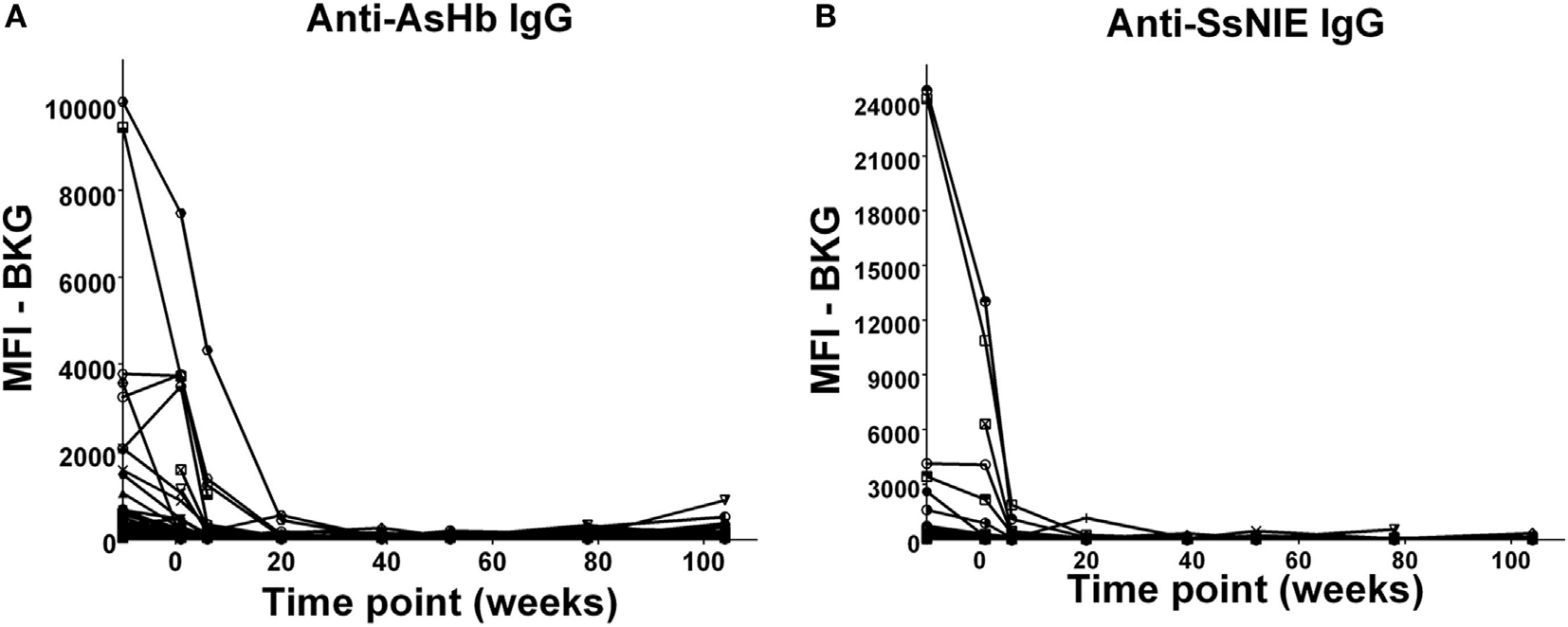
Time course of IgG antibody responses to *Ascaris suum* hemoglobin (AsHb) **(A)** and SsNIE **(B)** among infants from birth to 2 years. *x*-Axis shows time of sample collection, calculated from birth in weeks. Maternal antibody response is shown at −10 weeks. Neonatal first bleed is shown at 1 week and subsequent bleeds are at 6 weeks and 5, 9, 12, 18, and 24 months. MFI, median fluorescence intensity; BKG, background.

**Figure 5:**
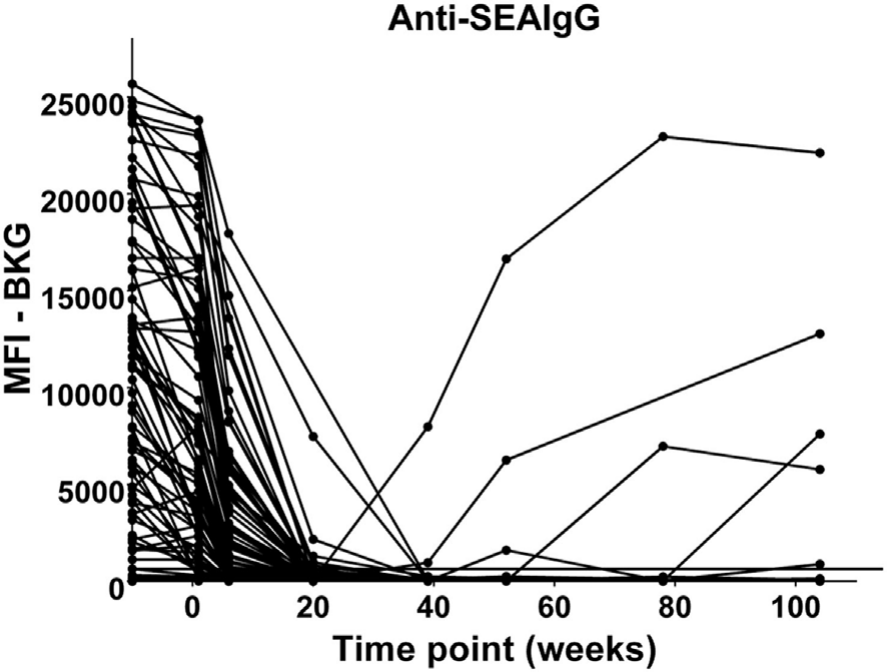
Time course of IgG antibody responses to soluble egg antigen (SEA) of *Schistosoma mansoni* among infants from birth to 2 years. *x*-Axis shows time of sample collection, calculated from birth in weeks. Maternal antibody response is shown at −10 weeks. Neonatal first bleed is shown at 1 week and subsequent bleeds are at 6 weeks and 5, 9, 12, 18, and 24 months. MFI, median fluorescence intensity; BKG, background. Horizontal line shows optimal cutoff (267) for positive antibody responses to *S. mansoni* SEA.

In the relatively few neonates who demonstrated anti-AsHb or anti-SsNIE antibody responses, following decline of maternal antibody, none of the children exhibited IgG antibody responses to these antigens during their first 2 years of life (Figures 4A,B). However, following the waning of maternal antibodies to SEA, 4 of the 65 children for whom there were specimens at 2 years of age (6%) were seen to have a vigorous IgG response to SEA, indicative of having acquired *S. mansoni* infection (Figure 5). At least two of these children had strong anti-SEA IgG responses by 1 year of age. Once generated, the anti-SEA responses of the four children remained stable or increased over time.

**Figure 6:**
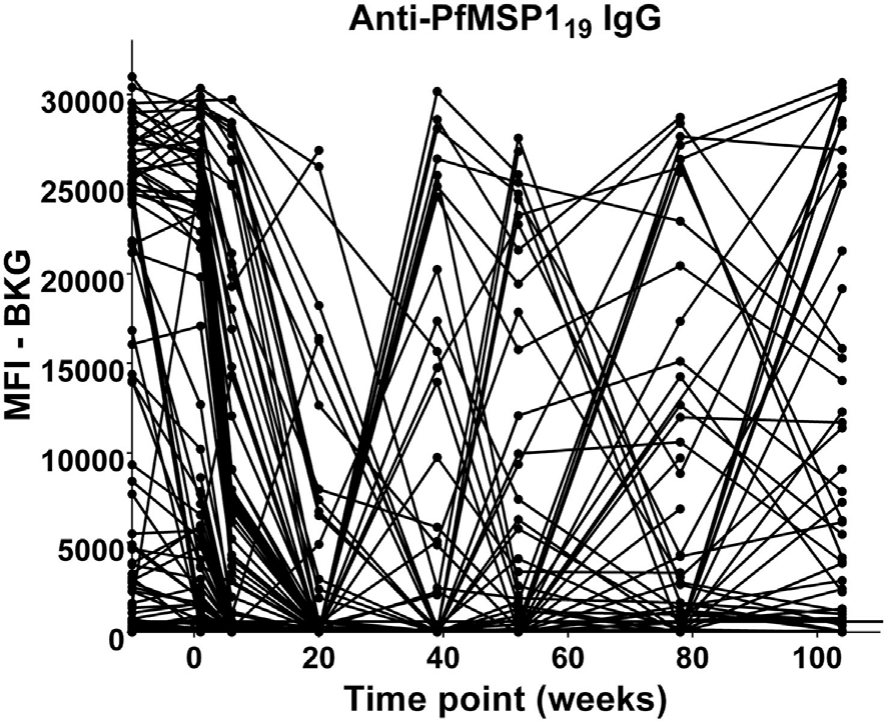
Time course of IgG antibody responses to PfMSP1_19_ among infants from birth to 2 years. *x*-Axis shows time of sample collection, calculated from birth in weeks. Maternal antibody response is shown at −10 weeks. Neonatal first bleed is shown at 1 week and subsequent bleeds are at 6 weeks and 5, 9, 12, 18, and 24 months. MFI, median fluorescence intensity; BKG, background. Horizontal line shows optimal cutoff (547) for positive antibody responses to PfMSP1_19_.

The anti-PfMSP1_19_ patterns of responses after the decline of maternal antibody are presented in Figure 6. The responses of children between 9 months and 2 years of age fall into four categories: (A) no positive response (22 children); (B) a single positive response episode with negative levels on either side (15 children); (C) a positive response, followed by a negative level, followed by a positive response (5 children); and (D) two to four positive responses in consecutive bleed periods (33 children). Therefore, 53 of the 75 (70%) children followed through these time points had at least one positive anti-PfMSP1_19_ episode and most of these had two or more, with many positive at consecutive time points.

## Discussion

### The Impact of Gestational Schistosomiasis on a Child’s Vaccine Responses

The question of whether a schistosome infection impacts vaccine responses continues to be of potential public health importance. One aspect of this question concerns children and adults being immunized while infected with schistosomes. This has been addressed in multiple studies, with mixed results. In some cases, it seems that initial responsiveness to hepatitis B vaccine (a primary response) and tetanus toxoid (a secondary response) is not impacted in young adults, but that the levels of their responses tend to wane earlier if they harbored schistosomes when immunized, perhaps indicating that those with schistosomiasis need to be boosted more regularly to maintain protective levels against these pathogens (29). Other studies in adults and children have not observed any significant impact by schistosome infections on vaccine responses (30, 31), while some smaller, earlier studies did report a negative impact (32).

The current study was designed to ask whether being born of a mother who had schistosomiasis during a child’s gestation altered their ability to respond to KEPI vaccines against tetanus, diphtheria, and measles. One potential confounder that should be mentioned is that we determined a mother’s schistosomiasis status based on the Kato Katz thick-smear stool assay, which is known to lack sensitivity in low intensity infections. We did assay three stool samples on three different days, using two slides per stool specimen, but we cannot rule out that some of our *S. mansoni* egg-negative mothers may have had very light, undetected, *S. mansoni* infections. The question of whether a pregnant woman’s schistosomiasis status influences the responsiveness of their child to such vaccines has been studied previously (2, 33–36), again with mixed results. In some studies a mother’s schistosomiasis had no impact on vaccine responses (30, 31, 36–38), while in others it did (4, 33, 34). In the current study, we did not see an impact of a mother’s schistosomiasis status on anti-tetanus or anti-diphtheria responses, but as a group at 2 years of age, children born of mothers with schistosomiasis responded with lower mean antibody levels to measles immunizations and proportionally fewer of them had levels that were above our assay cutoff. Our cutoff is estimated to correlate with protective levels of anti-measles antibodies (Figures 1 and 2). This result would seem to conflict with that of Webb, et al., 2011 (37), but in that report anti-measles responses were evaluated at 1 year of age, only 3 months after immunization, while we measured anti-measles antibody at 2 years of age (15 months after their measles immunization). Also, our observation in regard to diphtheria is in contrast to Malhotra et al. (34) who observed an impaired ability of children born to mothers with various helminth infections or malaria to develop protective levels of IgG antibodies to diphtheria antigen. Possible explanations for this difference include that their study was on the impact of *S. haematobium*, rather than *S. mansoni*, the children were followed every 6 months until 36 months of age, and the infection status of the mothers was based on both parasitologic and antibody data (i.e., anti-schistosome worm IgG4 levels).

### Antibody Responses of Children Born in Western Kenya to Parasite Antigens

The study design also allowed us to evaluate the maternal to child placental transfer of antiparasite antibodies to *S. mansoni, P. falciparum, A. lumbricoides*, and *S. stercoralis*, all pathogens that could be encountered by children in our study area. Analyses of the antibody levels in the paired plasmas of the mothers and their newborns confirmed the strong correlation anticipated between these levels, which is based on the active transport of IgG across the placental by FcRn (39) and results in neonate antibody concentrations that equal or even exceed maternal levels at delivery (40). Western Kenya is an area known to have relatively high levels of placental malaria, which could make this observation worthy of note. However, because only 7 of the 99 mothers studied had smear-positive malaria, the interpretation of whether placental malaria, which is best diagnosed by biopsy, interferes with IgG antibody placental transfer is not possible. However, several studies have associated placental malaria with decreased transfer of antibodies to measles, pneumococcus, and tetanus (41–43).

In addition, by following plasma levels of antiparasite antibodies of the newborns through to the age of 2 years, we were able to assess first the decrease of maternal antiparasite antibodies and then any rise in antiparasite antibodies that might likely reflect exposure to and/or acquisition of infection by those parasites. In this regard, it was clear that whatever antibodies the neonates received from their mothers against *A. lumbricoides* and against *S. stercoralis* were lost over the postnatal period and none developed in any of the 99 children over the first 2 years of their life (Figures 4A,B). This observation likely indicates a very low level of early life incidence of these two helminths in this area of Kenya. This is consistent with our previously reported very low levels of ascariasis in schoolchildren in the area (44) and in surrounding regions (45).

Children’s responses to SEA, indicative of infection by schistosomes, demonstrate that some children had acquired schistosomiasis as early as 1 and 2 years of age (Figure 5). This observation is in complete agreement with what is now widely accepted, i.e., that the incidence of schistosomiasis in preschool children is higher than previously thought (46, 47), and that preschool aged children (PSAC) are at risk of schistosomiasis-associated morbidity (48–50). Our observation of anti-SEA responses early in life in this area is also consistent with that of Won et al. (18). Although the need to treat PSAC is now clear, PZQ tablets are large and bitter tasting, making it a challenge to conduct MDA in this group. Therefore, it is worth noting that there is an active international public–private consortium working to develop a pediatric formulation of PZQ and it is now undergoing clinical trials (51–53).

Following the decline of maternal anti-PfMSP1_19_ antibodies, between 9 months and 2 years 53 of 75 children presented at on time or another with positive anti-PFMSP1_19_ responses. 15 children had a single episode of positive levels of anti-PfMSP1_19_ IgG, 5 had multiple episodes separated by periods of negative levels, and 33 were anti-PfMSP1_19_-positive at 2, 3, or 4 consecutive time points (Figure 6). There is evidence of *in utero* sensitization of both T and B lymphocytes to PfMSP-1 (54). However, based on the low level of malarial smear positivity of the mothers in our study and the decline in most children to baseline of the anti-PfMSP1_19_ responses by 20 weeks, we conjecture that the anti-PfMSP1_19_ IgG episodes we detected between 9 months and 2 years are indicative of recently acquired *P. falciparum* infections (55, 56). Thus, based on antibody responses, only 22 out of 75 (29%) children never experienced a *P. falciparum* infection by the age of 2 years. While this might be expected in this area, which is holoendemic for *P. falciparum*, this documentation is of interest in the face of multiple malaria control programs in the area (57).

In conclusion, our data indicate that the presence or absence of *S. mansoni* in a pregnant woman does not significantly impact her neonate’s ability to respond to immunizations against tetanus or diphtheria. In addition, the data suggest that while many neonates born of mothers with *S. mansoni* respond reasonably well to their 9-month measles immunization, some do not. The antibody levels against measles N-protein and the proportion of children who were IgG positive are statistically lower for children born to *S. mansoni*-infected mothers compared to children whose mother was *S. mansoni*-negative. This may mean that to get optimum effectiveness of measles immunization, treating pregnant women for their schistosomiasis should be emphasized, which would be beneficial for both the mother and child. Additional studies of measles vaccine efficacy need to be done to determine whether children in schistosomiasis endemic areas may benefit from receiving an extra measles vaccine booster dose.

## Data Availability

All relevant data are presented within the article.

## Ethics Statement

The research protocol was approved by the Scientific Steering Committee of the Kenya Medical Research Institute (SSC-KEMRI), KEMRI/Scientific Ethical Review Unit (Protocol No. 2303) and the Institutional Review Board at the University of Georgia (Protocol No. 00004091). The Centers for Disease Control and Prevention (CDC) also reviewed the protocol; CDC personnel were considered not to be engaged because they had no contact with study participants or access to personal identifiers. All subjects provided written informed consent in accordance with the Declaration of Helsinki.

## Author Contributions

DC, WS, EM, and DK participated in the study design. EM, JO, and BO led the enrollment of subjects and the collection of specimens, and HK, EM, SN, EN, and BO performed the laboratory assays of the specimens. NK and JP analyzed the data, and DC, WS, NK, JP, and BO wrote the manuscript. All authors participated in reviewing and editing the manuscript and concurred with the final manuscript.

## Conflict of Interest Statement

The authors declare that the research was conducted in the absence of any commercial or financial relationships that could be construed as a potential conflict of interest.
